# Lymphedema after saphenous harvesting for coronary artery bypass surgery: case report and literature review

**DOI:** 10.1186/s12872-024-03712-6

**Published:** 2024-01-12

**Authors:** Seyed Mohsen Mirhosseini, Masood Soltanipur, Hossein Yarmohammadi, Mahdi Rezaei, Zahra Sheikhi

**Affiliations:** 1 Cardiovascular Research Center, Shahid Beheshti University of Medical Sciences, Tehran, Iran; 2 Medical Students Research Committee, Shahed University, Tehran, Iran; 3 Quality of Life Department, Breast Cancer Research Center, Motamed Cancer Institute, ACECR, Tehran, Iran; 4 Integrative Oncology Research Group, Breast Cancer Research Center, Motamed Cancer Institute, ACECR, No.146, South Gandi Ave, Vanak Sq, Tehran, 1517964311 Iran

**Keywords:** Lymphedema, Saphenous harvesting, Coronary artery bypass, Quality of life

## Abstract

Different causes have been described for secondary lymphedema as reported in this article. A 75-year-old man was diagnosed with lymphedema about one decade after saphenous harvesting for coronary artery bypass surgery. It took two years for him to find out his diagnosis and receive the proper treatment. After standard complete decongestive therapy, his volume and pain decreased and his quality of life was improved, especially its physical aspect. It is important to recognize the possibility of lymphedema development after saphenous harvesting among patients undergoing coronary artery bypass surgery to prevent significant disturbance of quality of life with timely management.

## Introduction

Coronary artery bypass graft (CABG) surgery serves as the established modality for a considerable number of patients afflicted with complicated, multi-vessel coronary artery disease [1]. Saphenous vein grafts are the conduits that are most commonly used in CABG [2]. Complications frequently documented after saphenous vein grafts include dermatitis, cellulitis, neuropathy, chronic non-healing wounds, lymphocele, and lymphedema. In the majority of CABG procedures, the occurrence of these complications seldom requires surgical intervention and presents a minor concern [3]. Lymphedema is characterized as a specific manifestation of tissue edema caused by an increase of lymphatic fluid accumulation within the interstitial space, which occurs as a consequence of weakened lymphatic drainage. Secondary lymphedema is obtained as a consequence of an underlying trauma, infection, and, surgical procedure [4]. Lymphedema does not have a cure and the standard approach includes manual lymphatic drainage (MLD), physical activity, bandaging, and, skin-care or as also called complete decongestive therapy (CDT) [5]. In this case report, we present a 75-year-old male who has undergone CABG surgery and has developed lower limb lymphedema after 11 years.

## Case presentation

**Fig. 1:**
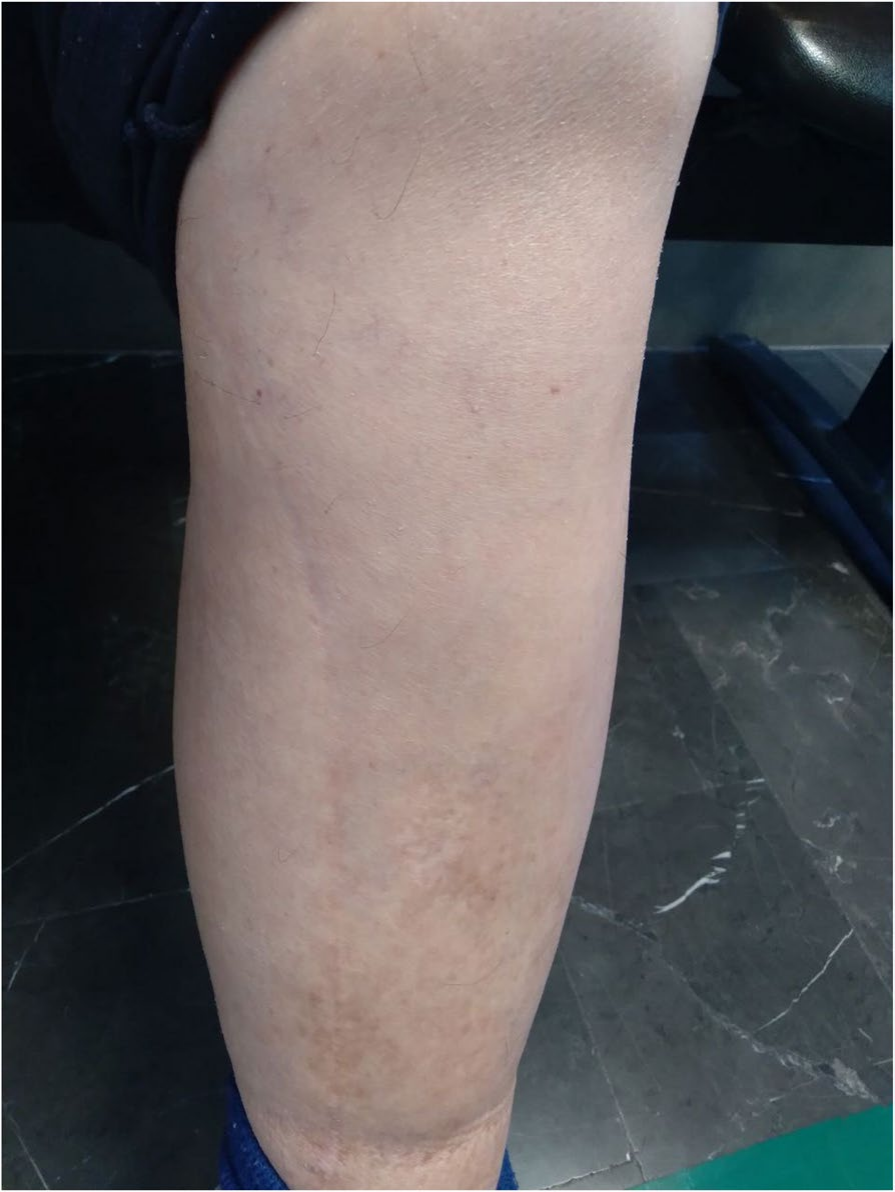
The scar of saphenous harvesting on the left edematous lower limb

**Fig. 2:**
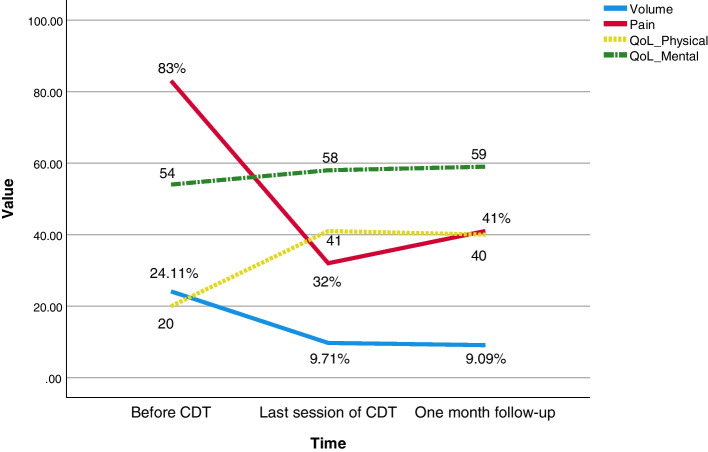
Changes in volume difference, pain, and QoL physical and mental domains

The case was a 75-year-old man who was seeking consultation in our lymphedema clinic due to lower limb edema and pain. His past medical history included atrial fibrillation, degenerative joint disease, and, CABG at 13 years ago. He did not mention any trauma or surgical history of his lower limb after saphenous harvesting. Drug history included rosuvastatin, furosemide, and, warfarin. His lower limb edema started two years ago and several episodes of cellulitis have occurred. During the first year, he was treated with diuretics with the suspicion of leg edema caused by congestive heart failure despite his stable ejection fraction over the past years. The diuretic consumption has not been effective and after almost one year, he started experiencing pain in his lower limb. He tried many different specialties yet did not even find out the name of his condition. In our center, he was frustrated and complaining of his pain which clearly had disturbed his quality of life (QoL). At physical examination, non-pitting edema of the left lower limb was evident with a positive Stemmer sign. Fibrotic skin changes were also observed as well as the scar from saphenous harvesting surgery as presented in Fig. 1. The difference in limb volume was about 24% using tape measuring. His BMI was 27.7 kg/m^2^ and the rest of his examination was unremarkable. He had brought some medical records with him such as blood tests and echocardiography which none showed any unexpected abnormality according to his past medical history. He also had four Doppler ultrasonography during the past two years the last one was about three months ago and all were normal. There was no evidence of thrombosis and both arterial and venous systems of the upper and lower left limb were healthy. As a part of the initial assessment of the first visit to the clinic, he was assessed for possible psychosocial problems. He was desperate to be referred over and over and yet not able to sleep through the night because of the pain. He said “I cannot sleep, I cannot play with my grandchild, and, I cannot even wear my clothes. This leg hurts and sometimes it even gets red and burns. I stayed in the hospital for its redness. At first, it was my heart, then my leg. I’m exhausted”. He then continued “I have found this clinic on the internet. I have tried all kinds of treatments. You are my last hope.” Despite being miserable, he did not seem to have depression or other mental disorders based on an interview with the physician. He was informed that he was suffering from lymphedema. After educating him about this condition, CDT was initiated by the physiotherapist in our center. CDT consisted of two phases; phase I which included MLD, compression bandaging, exercise, and skin care five days per week for four weeks. MLD at each session took 40 minutes based on the Dr. Vodder method [6]. And, phase II consisted of self-care at home to maintain clinical achievements in phase I [7]. The initial sessions of treatment were challenging since the patient did not have much compliance with the treatment. However, by educating him about lymphedema and taking the time to address all his questions and concerns, the treatment was completed with favorable results. His volume reduced from 24.11% to 9.71% and 9.09% after the last session of phase I and at the one-month follow-up respectively. The pain was assessed based on a visual analog scale and was 8%, 32%, and, 40% respectively. His QoL was also assessed using the SF-12 questionnaire which contains two physical and mental categories. The mental score was acceptable even before the treatment but the physical score was significantly low mostly due to pain. Both mental and physical scores of QoL were improved after CDT and remained at an acceptable level after one month of follow-up as shown in Fig. 2.

## Discussion and review of the literature

### Lymphedema is an underrecognized complication of saphenous harvesting

Lymphedema is a chronic disease with a significant impact on QoL. The prevalence of lymphedema has not been fully studied since its etiologies are very different, however, lymphatic filariasis and cancer-related lymphedema affect many individuals globally [8, 9]. Theoretically, any injury to lymphatic circulation may initiate the inflammatory pathophysiology of secondary lymphedema which will lead to fibro-adipose changes in the tissue at the late stages of the disease [10]. Lymphedema after saphenous surgery rarely has been reported in the literature. Heitink et al. reported a 62-year-old female who developed lymphedema one year after saphenous harvesting for CABG [11]. Although only the harvested leg was clinically swollen, interestingly lymphoscintigraphy showed bilateral diminished uptake. This finding may suggest that the underlying problems with lymphatic drainage before the surgery contribute to lymphedema presentation after saphenous harvest. The diagnosis of lymphedema in our clinic is made clinically based on the International Society of Lymphology guidelines and the American Venous Forum, American Vein and Lymphatic Society, and the Society for Vascular Medicine expert opinion consensus on lymphedema diagnosis. Therefore, unfortunately, we did not have such data on his lymphatic circulation using lymphoscintigraphy [5, 12]. In addition to lymphoscintigraphy, other new imaging modalities such as magnetic resonance imaging lymphangiography are among the most accurate diagnostics for lymphedema, however, these imaging are expensive, invasive, or not always available [13, 14]. Limited centers perform lymphoscintigraphy and the costs of this imaging may not be affordable for many patients. Therefore, the diagnosis of lymphedema is made clinical in our limited resources setting [15]. Nevertheless, the case report by Heitink et al. showed diminished lymphatic uptake one year after saphenous harvest [11]. In our case, this man’s lymphedema presented about a decade after saphenous harvesting for CABG which makes it different than the case reported by Heitink et al.

The long interval between lymphatic injury and lymphedema presentation has been reported among BCRL patients. The prospective study of Ribeiro Pereira et al. pointed out that lymphedema may present even after ten years [16]. Similarly, other studies have reported lymphedema presentation at 10, 20, and even 30 years after surgery among breast cancer survivors [17–19]. This risk is well-recognised in clinical practice and many activities have been proposed to be avoided lifelong such as venectomy from the limb at the side of the surgery [20]. Although this should always be reminded that one case report would never bring any causality or certainty and still more evidence is needed, yet this case might bring more attention to the long-term care and follow-up of CABG patients. Additionally, the study by Belczak et al. reported delayed lymphedema after saphenous harvesting. In this study, 44 patients were assessed using water displacement and lymphoscintigraphy after a mean interval of 46 months [21]. It seems that even without direct injury to lymphatic circulation during saphenous harvesting, disturbed venous circulation might play a role in making patients susceptible to lymphatic complications such as lymphedema. This hypothesis could also be supported by the association of chronic venous disease with lymphedema in the literature [22, 23]. As far as our search showed, there were no other case reports of lymphedema after saphenous harvesting for CABG than the case reported by Heitink et al. [11]. Additionally, the study of Sharquie et al. reported that seven out of 100 patients complained of lymphedema after a median of five months follow-up, however the diagnosis of lymphedema has not been described and limited data were available in this regard [24]. Nevertheless, it seems that both lymphatic and venous circulation could affect one another and lymphedema is a potential complication among patients undergoing CABG.

### From neglecting lymphedema to the patient’s QoL

Lymphedema results in a variety of symptoms such as pain, paraesthesia, heaviness, and, edema [25, 26]. The size difference of limbs makes daily life difficult and also might be disfiguring for the patient [27, 28]. Lower limb lymphedema also has been reported to negatively impact physical activity and to be associated with sleep disturbances which reduce the quality of sleep and QoL [29, 30]. The role of pain in distressing QoL is well-documented in the literature and depression and anxiety disorders are clinically important comorbidities among lymphedema patients [31]. In our case, the man has been experiencing many of the mentioned problems, however, there is one aspect of this case that needs to be highlighted: It took two years for him to find out the name of his disease. This frustration has imposed a significant burden on him. He willingly tried many different complementary and alternative therapies with no effect. Diuretics also have been tried with no improvement in his situation. It is important to distinguish lymphedema from possible differential diagnoses such as congestive heart failure, chronic venous diseases, and, lipedema [32]. Although the diagnosis could be challenging at his age accounting for his cardiovascular history, some clinical signs and symptoms could be useful to make such a distinction. The unilateral limb edema with a positive Stemmer sign and a normal Doppler ultrasonography is strongly suggestive of lymphedema. Making the right diagnosis is related to the knowledge of healthcare providers and there is a substantial gap in this field [33, 34]. Lymphedema is somehow neglected in medical research and education and the outcome of this neglect would reflect on clinical practice [35–37]. Knowledge of lymphedema is essential to take preventive measures and reduce the risk of lymphedema development following saphenous harvesting surgery. New research is directed to minimally invasive surgical techniques for saphenous harvesting. The study by Cisowski et al. compared three less invasive surgical techniques with the open surgery of saphenous vein harvesting. In this prospective randomized trial, endoscopic harvesting was used by different techniques as minimally invasive surgery for saphenous harvest. After seven days post-operation, the number of patients with edema was significantly lower among three arms compared to the open surgery. Other outcomes such as wound healing or pain were also better among patients who received endoscopic surgery [38]. Šimek et al. reported similar results in their prospective trial comparing minimally invasive and endoscopic great saphenous harvesting. Lymphatic discharge was significantly lower among the endoscopic group at seven days post-operation. Also, residual edema was significantly lower both at seven and three months follow-up after endoscopic surgery [39]. Additionally, early rehabilitation to restore lymphatic drainage is another means to prevent lymphedema following saphenous harvesting surgery but unfortunately, his lymphedema was not diagnosed and he was not referred to the specialized center for lymphedema management. Interestingly, he has performed four Doppler ultrasonography during the past two years which all were normal. These are all indicators of direct and indirect costs of lymphedema which burden healthcare systems. Such costs also could play a role in disturbed QoL [40, 41]. Recognizing lymphedema in the field of cardiovascular surgery is one way to prevent such unnecessary costs.

## Conclusion

Saphenous harvesting could be a risk factor for lymphedema development among patients undergoing CABG surgery. Recognizing this complication could help to prevent significant disturbance in QoL with timely intervention. Little is known about the pathophysiology of lymphedema, specifically the contribution of venous circulation. Therefore the interaction of these two lymphatic and venous circulations and their impact on one another could be an interesting research topic of the future.

## Data Availability

Data are available based on a request from the corresponding author.
